# Pilot implementation to assess the feasibility and care team impact of an app-based interactive care plan to remotely monitor breast cancer survivors

**DOI:** 10.1007/s11764-021-01136-1

**Published:** 2022-02-02

**Authors:** Daniela L. Stan, Jonathan W. Inselman, Jennifer L. Ridgeway, Kaley N. Johnson, Laura A. Christopherson, Samantha M. McColley, Julie K. Brown, Sarah A. Phillips, Summer V. Allen, Jennifer K. Hazelton, Kathryn J. Ruddy, Tufia C. Haddad

**Affiliations:** 1 General Internal Medicine, Mayo Clinic, 200 First Street SW, Rochester, MN 55905 USA; 2 Kern Center for the Science of Health Care Delivery, Mayo Clinic, 200 First Street SW, Rochester, MN 55905 USA; 3 Center for Digital Health, Mayo Clinic, 200 First Street SW, Rochester, MN 55905 USA; 4 Family Medicine, Mayo Clinic, 200 First Street SW, Rochester, MN 55905 USA; 5 Medical Oncology, Mayo Clinic, 200 First Street SW, Rochester, MN 55905 USA

**Keywords:** Interactive care plan, Cancer survivorship, Self-management, Remote patient monitoring, Symptom control, Mobile application

## Abstract

**Purpose:**

To assess the feasibility of an app-based, electronic health record (EHR)-integrated, interactive care plan (ICP) for breast cancer (BC) survivors.

**Methods:**

A single-arm pilot study was conducted with female BC survivors. ICP tasks included quarterly quality of life (QOL) questionnaire; monthly assessments of fatigue, insomnia, sexual dysfunction, hot flashes, and recurrence symptoms; and daily activity reminders. Embedded decision trees escalated recurrence symptoms to providers. On-demand education was available for self-management of treatment-related toxicities. The primary objective was to assess patients’ engagement with ICP tasks against feasibility thresholds of 75% completion rate. Secondary objectives were evaluation of the system’s functionality to track and escalate symptoms appropriately, and care team impact measured by volume of escalation messages generated. We report preliminary results 6 months after the last patient enrolled.

**Results:**

Twenty-three patients enrolled August to November 2020. Mean age was 50.1 years. All patients engaged with at least one ICP task. The monthly average task completion rates were 62% for the QOL questionnaire, 59% for symptom assessments, and 37% for activity reminders. Task completion rate decreased over time. Eleven of 253 symptoms and QOL questionnaires (4.3%) generated messages for care escalation.

**Conclusion:**

Implementation of an app-based, EHR-integrated ICP in BC survivors was feasible and created minimal provider burden; however, patient engagement was below the feasibility threshold suggesting that changes may enhance broad implementation and adoption.

**Implications for Cancer Survivors:**

An ICP may facilitate remote monitoring, symptom control, and recurrence surveillance for cancer survivors as strategies to enhance patient engagement are applied.

**Supplementary Information:**

The online version contains supplementary material available at 10.1007/s11764-021-01136-1.

## Introduction

Breast cancer (BC) has recently surpassed lung cancer to become the most diagnosed malignancy in women worldwide, with an estimated 2.3 million new cases diagnosed in 2020 [1]. Currently, there are 7.8 million BC survivors globally, making it the most prevalent cancer; moreover, BC is responsible for a larger share of lost disability-adjusted life years around the world than any other type of cancer [2]. The population of BC survivors is expected to grow substantially by 2030 [3], due to an aging population, increased cancer screening in racial/ethnic minority populations, and improved technologies for cancer detection [4]. The longitudinal follow-up care of BC survivors is complex, resource intensive, and in conflict with the imminent projected shortage of multidisciplinary cancer treatment specialists [5–7].

Currently, survivorship care consumes a substantial proportion of the typical oncology clinic workday, taking time from patients with new diagnoses and in need of active treatment decisions and monitoring. The burden and complexity of BC survivor care is escalating, and the current model of care is unfit to sustain this growth. Moreover, BC survivors feel “lost in transition” after they complete the intensive multi-modality treatment as follow-up care can be duplicative and fragmented when multiple teams, rehabilitation specialists, and primary care providers are all co-managing with suboptimal communication and coordination [8]. This creates a mismatch between the intense needs of these patients in the immediate post-primary treatment period, when they are not only at risk for recurrence but also at risk for persistent treatment-related toxicities, and the availability of oncological care.

There is great need for new models of care delivery for cancer survivors [9, 10], as well as “well-informed and engaged patients at the center of care” [9, 11]. It has become a clear social and economic imperative to create a novel, high-value, low cost care model for the long-term management and engagement of cancer survivors with their multidisciplinary care team.

Technology-based tools for symptom and physiologic monitoring, such as telehealth and remote patient monitoring, have become prominent in the last decade for chronic condition management [12, 13]. Most of the telehealth studies worldwide targeted patients with cardiovascular, pulmonary, and endocrine diseases; however, there is a paucity of studies in oncology [14]. The few cancer-related studies have involved electronic patient-reported outcomes (ePROs) for monitoring of systemic therapy-related side effects [15, 16] and cancer progression in those with advanced disease [17, 18]. Cancer patient engagement with telehealth solutions has been demonstrated in a feasibility study evaluating utilization and PRO capture by a mobile application of perioperative education and support for breast cancer surgery [19] and a randomized controlled trial of a telerehabilitation tool for patients with advanced cancers [20]. However, there is a need to study the feasibility of telehealth and virtual care tools to address the complex needs of cancer survivors, including management of treatment-related toxicities, surveillance of QOL and symptoms associated with recurrence, and promotion of wellness and healthy life habits.

Our institution deployed survivorship care plans in 2013 as routine care, but the plans lacked actionable guidance to foster self-management and interactive engagement between patients and members of their care team. To address the unmet needs of BC survivors and transform the survivorship care delivery model at our institution, we collaborated with our Center for Innovation on a “Living Beyond BC” project from 2014 to 2015. During this 2-year period, interviews and focus groups were conducted with patients, as well as physicians, advanced practice providers, and nurses from multiple disciplines (medical oncology, radiation oncology, breast and plastic surgery, internal medicine breast specialists, primary care) to understand survivors’ unmet needs and barriers to long-term follow-up with primary care. Rapid experiments and paper prototyping of a web- or app-based solution were completed and informed development of seven patient-centered design principles and a conceptual model for a BC survivorship interactive care plan (ICP). It was hypothesized that the ICP could make the essential elements of a static survivorship care plan into an actionable and engaging guide for patients, and furthermore, that oncology-led remote monitoring services enabled by the ICP could facilitate earlier transitions to primary care provider-led longitudinal follow-up.

**Fig. 1:**
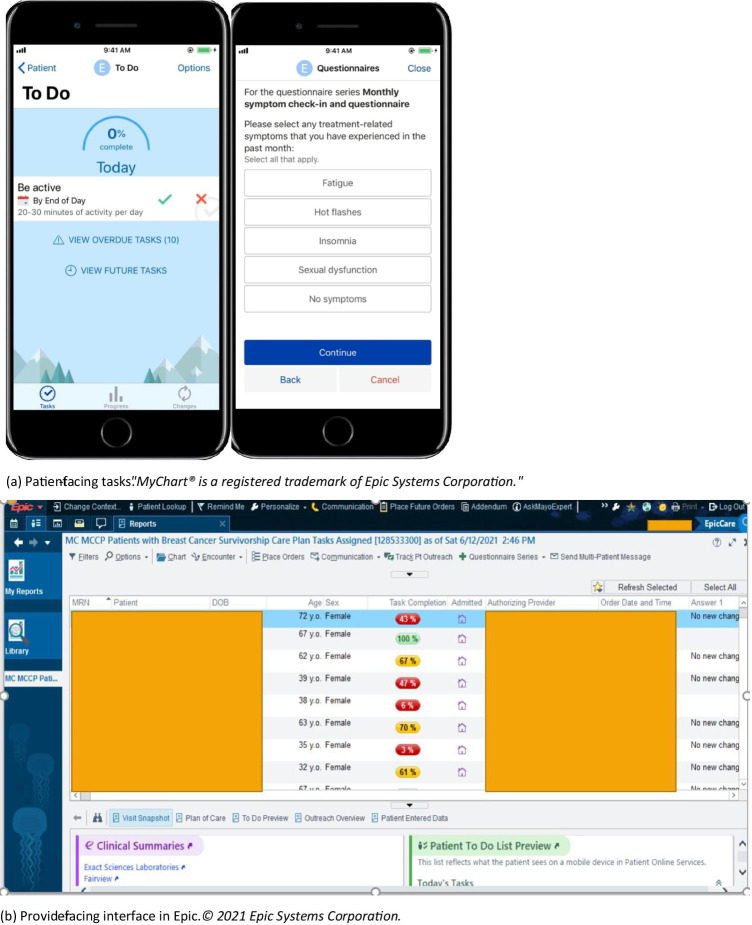
Patient-facing screenshots of the ICP tasks (**a**) and provider-facing ICP report in Epic (**b**). **a** Patient-facing tasks. “MyChart® is a registered trademark of Epic Systems Corporation.” **b** Provider-facing interface in Epic. © 2021 Epic Systems Corporation

In collaboration with our institution’s Electronic Health Record (EHR) vendor (Epic), our team of business analysts, product specialists, health system engineers, and clinicians developed the ICP (referred to as Epic’s MyChart® Care Companion). The ICP is a fully EHR-integrated tool, embedded within our Mayo Clinic mobile application, which can be accessed through a patient’s mobile device via their patient online services (portal) account. Software developers from Epic designed the software and key functionality that serves as the technology platform. This tool has both patient- and provider-facing interfaces (Fig. 1), allowing for seamless integration of patients’ responses into the EHR where they can be visualized in real time by the oncology care team.

Our primary objective was to assess the feasibility of the ICP with BC survivors. Secondary objectives were to evaluate the ICP functionality to track and act upon self-reported symptoms as needed, and to assess the care team impact of care escalation messages generated by the ICP. User engagement, like that in our “Living Beyond BC” project, is critical to developing interventions that meet the needs of patients and care teams. An important next step is assessment of whether the app-based ICP intervention can be feasibly implemented in clinical workflows and patients’ daily lives before expanding to larger deployment and study [21]. It may also identify barriers to implementation and potential strategies to address them. Herein, we report preliminary results of our feasibility pilot study at 9 months following activation and 6 months after the last patient enrolled.

## Methods

### Study design and patient recruitment

A single-arm pilot feasibility study in female BC survivors was conducted within the breast oncology practice of a large academic medical center. Patients 18 years of age and older, diagnosed with stage 0–3 BC who had completed definitive breast surgery, and as appropriate, systemic chemotherapy and radiotherapy, for BC treatment within the prior 12 months, were eligible to participate. Other inclusion criteria included access to a mobile device (smart phone or tablet), an established patient online services (portal) account, and long-term follow-up planned at Mayo Clinic. Patients who were unable to provide informed consent, speak or read English language, and participate in mild activity or who resided in the European Union (due to General Data Protection Regulations rules) were excluded. Ongoing treatment with adjuvant endocrine, bisphosphonate, and HER2-directed therapy during the study period was allowed.

Members of the research team or the care team screened the list of patients scheduled for a BC Survivorship Consult, which occurs per standard practice within 12 months following completion of active BC treatment. Those interested and meeting the inclusion criteria were subsequently informed of the study requirements, potential risks and benefits, and standard care options. Those consenting to participate were subsequently enrolled after their BC Survivorship Consult visit. The care provider then placed an order for the BC survivorship ICP in Epic.

This study was approved by the Mayo Clinic Institutional Review Board (IRB #19–002,448).

### Intervention

The BC survivorship ICP is EHR-integrated with care plan tasks delivered through the Mayo Clinic mobile app. The app generates mobile device notifications to the patient when ICP tasks are due (Supplemental Fig. 1). The tasks include monthly ePRO assessments of four treatment-related toxicity symptoms (fatigue, insomnia, sexual dysfunction, and hot flashes); monthly surveillance of signs and symptoms associated with BC recurrence; quarterly QOL; and daily reminders to be physically active. The ICP provides symptom(s)-specific educational materials, upon demand, for self-management of reported toxicities with different educational contents delivered every month. In addition, tobacco cessation and mindfulness education modules are provided at baseline and every 6 months. The patient education content that is made available to patients (either scheduled or in response to a symptom reported) is indexed within the Epic MyChart Care Companion “Education Library” for patients to access beyond its initial delivery.

The ICP contains embedded logic which based on a patient’s response to symptom assessments and questionnaires may escalate care as an automated message sent to the research care team through the EHR. The EHR inbox (Epic “In Basket”) is monitored at least twice a week by two research coordinators, including a nurse. Escalation occurs when a patient reports any of the symptoms concerning for BC recurrence or a score ≥ 4 (on a scale from 1 to 5) on the anxiety and/or depression domains of the QOL tool, or when being dismissed from a Mayo Clinic hospital (as the ICP is paused during a hospital admission). For clinically relevant escalations, the research team then sends a message to the patient and the BC care team regarding the concern raised through the app-based surveillance. The care teams are comprised of internal medicine and medical oncology providers who follow the BC survivors longitudinally. The messages are labeled as “research ICP” in the “reason for communication” in the EHR, to be trackable by the research team.

For easier tractability and visualization of patients’ interaction and answers in the ICP, we developed an ICP dashboard containing separate visualizations for the treatment-related toxicity symptoms (including the requests for education), recurrence symptoms, activity task, and Patient-Reported Outcomes Measurement Information System (PROMIS) scores.

The BC survivorship ICP is designed as a 12-month plan. After completion of the last task within the app at month 12 and the completion of other end-of-study questionnaires, study participation is discontinued. Patients could opt out of study participation at any time.

### Tools

The ePROs assess the burden of the specific symptom through single item linear analog scale assessment (LASA) for fatigue, insomnia, sexual dysfunction, and hot flashes. The patient rates her discomfort on a scale of 0–10, with higher scores denoting worse symptoms. Studies suggest that single items may be sufficient when only a global impression of QOL is needed. LASA items have been shown to be valid and clinically appropriate [22, 23] and have been used to evaluate patients with cancer [24].

QOL was assessed through the Patient-Reported Outcomes Measurement Information System (PROMIS) 29 questionnaire, available in the Epic toolbox. The PROMIS-29 is quickly becoming a standard for Patient-Reported Outcomes (PRO) research and practice [25]. PROMIS tools have a strong construct validity and feasibility [26, 27]. PROMIS-29 includes seven domains (Physical Functioning, Anxiety, Depression, Fatigue, Sleep Disturbance, Social Functioning, and Pain Interference), each with four items, rated 1–5. In addition, there is a question on pain intensity using a single 0–10 numeric rating scale. The PROMIS measures are scored on a T score metric in which 50 is the mean of a general US adult reference population and 10 is the standard deviation (SD) of that reference population. Higher scores denote more of the tested outcome.

### Outcomes

Our primary objective was to evaluate the feasibility of implementing the BC survivorship ICP in the oncology practice. There were three feasibility-related outcomes: (1) patient engagement, (2) ePRO, recurrence questionnaire, and QOL score tracking and actions, and (3) care team burden.

First, we assessed whether BC survivors engaged with the ICP by completing the tasks assigned. Second, we assessed whether the app could be used to track treatment-related toxicities, BC recurrence symptoms, QOL, and activity levels over time, and whether self-reported assessments would trigger appropriate follow-up actions. This included educational materials based on symptom scores and nurse triage for symptoms associated with BC recurrence or moderate to severe anxiety and/or depression scores. Third, we assessed the impact of the ICP implementation in the BC survivor clinical practice as measured by ICP-generated care escalation messages requiring nursing assessment and/or clinical intervention.

Due to a technical programming error, the app was unable to distinguish between the “I did it” and the “I did not do it” answers to the “Be active” daily reminder; thus, the frequency of the physical activity was not quantifiable. Instead, the frequency of patients interacting with the “Be active” task (whether they answered it or not) will be reported.

### Statistical methods

Since this is a single-arm pilot study of feasibility outcomes, there was no need for a power calculation. Analysis was performed according to the intention to treat principle, including all participants in the trial, regardless of whether they participated in the intervention. Baseline patient characteristics are reported using continuous values (means and standard deviations) and categorical values (frequencies and percentages).

For the primary outcome of questionnaire task completion rate (toxicity ePROs, recurrence symptom questionnaires, and QOL questionnaires), we assessed the total response rate of all questionnaires administered over the course of the study. The intervention was deemed feasible if there was a completion rate of ≥ 75%. The reminders task completion rate was similarly assessed. Task completion rate at baseline was compared to study end, using a paired *t* test.

For the secondary outcomes of the trackability of patients’ responses, we compared the patient’s answers visible in Epic with the patient’s answers in the ICP dashboard. To assess the actions that the ICP took in response to patients’ answers, we compared the number of automatic escalations received to the research In Basket to the number of recurrence symptoms plus anxiety/depression symptoms reported in the ICP dashboard. We also monitored the matching between toxicity symptoms levels and the education offerings and education usage in the ICP dashboard.

To assess the clinical care burden, our a priori feasibility threshold was that concerning monthly cancer surveillance symptoms, as well as worrisome anxiety and depression scores, would result in limited (< 10%) escalations to the BC care team. We assessed the clinical burden on the BC care team by tracking the number of escalations received by the research team plus the number of patient online messages and messages to the BC care team generated by the escalations, as documented in the EHR.

For sample size, to account for reliability and usage testing, we planned on analyzing 20 participants; anticipating a 10% attrition rate for early dropouts and screening failures, we aimed to enroll 23 participants. The analysis of quantitative data was performed as of the data lock on June 15, 2021. This timeline was based on time to enroll the 23 participants and being mindful of having enough time to assess the care team burden and make changes as needed. Analysis was completed using SAS 9.4 (SAS Institute, Cary NC).

## Results

### Patient engagement and task completion rates

From August 2020 through November 2020, we recruited 23 patients. The mean age was 50.1 years (range 30–75). The majority of patients were married and of non-Hispanic/LatinX ethnicity and white race. There was variability among patients by stage of disease, surgical choice, and receipt of adjuvant chemotherapy, radiation, and endocrine therapy (Table 1). Of the 23 participants, all engaged at least once during the study period with tasks assigned by the ICP, resulting in a 100% engagement rate at the first data collection timepoint, which was June 20, 2021. No patient asked to discontinue study participation or terminate the ICP.

**Table 1:** Baseline demographics and treatments information

	Total (*N* = 23)
**Age**	
Mean (SD)	50.1 (13.1)
Median (range)	51 (30,75)
**Race**	
Unable to provide	1 (4.3%)
White	22 (95.7%)
**Ethnicity**	
Central American	1 (4.3%)
Not Hispanic or Latino	21 (91.3%)
South American	1 (4.3%)
**Marital status**	
Married	21 (91.3%)
Single	2 (8.7%)
**Education level**	
High school or less	2 (8.7%)
Trade school or associate or some college	5 (21.7%)
Bachelors	8 (34.8%)
Masters or doctorate or professional degree	8 (34.8%)
**Stage of cancer**	
0	5 (21.7%)
IA	8 (34.8%)
IIA	6 (26.1%)
IIB	3 (13.0%)
IIIA	1 (4.3%)
**Surgical breast procedure**	
Bilateral mastectomy	12 (52.2%)
Lumpectomy	7 (30.4%)
Unilateral mastectomy	4 (17.4%)
**Surgical axillary procedure**	
SLN biopsy	19 (82.6%)
Axillary dissection	2 (8.7%)
None	2 (8.7%)
**Chemotherapy**	
No	14 (60.9%)
Yes	9 (39.1%)
**Radiation**	
No	12 (52.2%)
Partial breast	2 (8.7%)
Post-mastectomy	4 (17.4%)
Whole breast	5 (21.7%)
**Endocrine therapy**	
No	12 (52.2%)
Aromatase inhibitors	5 (21.7%)
Tamoxifen	6 (26.1%)
**Ovarian suppression**	
No	22 (95.7%)
Yes	1 (4.3%)
**Adjuvant IV or SQ Osteoclast inhibitors**	
No	22 (95.7%)
Bisphosphonate or RANKL	1 (4.3%)

The overall task completion rate (6-month average) to the monthly ePRO assessments for the four toxicity symptoms of interest (fatigue, insomnia, sexual dysfunction, and hot flashes) was 59%, with a gradual decrease from 78% at baseline to 48% by month 6 (*p* = 0.0078). This was identical with the task completion rate for the monthly questionnaire assessing symptoms of recurrence. The monthly “Perform a breast/chest wall examination” task completion rate declined from baseline with a monthly average of 12% (range 0–26%).

**Fig. 2:**
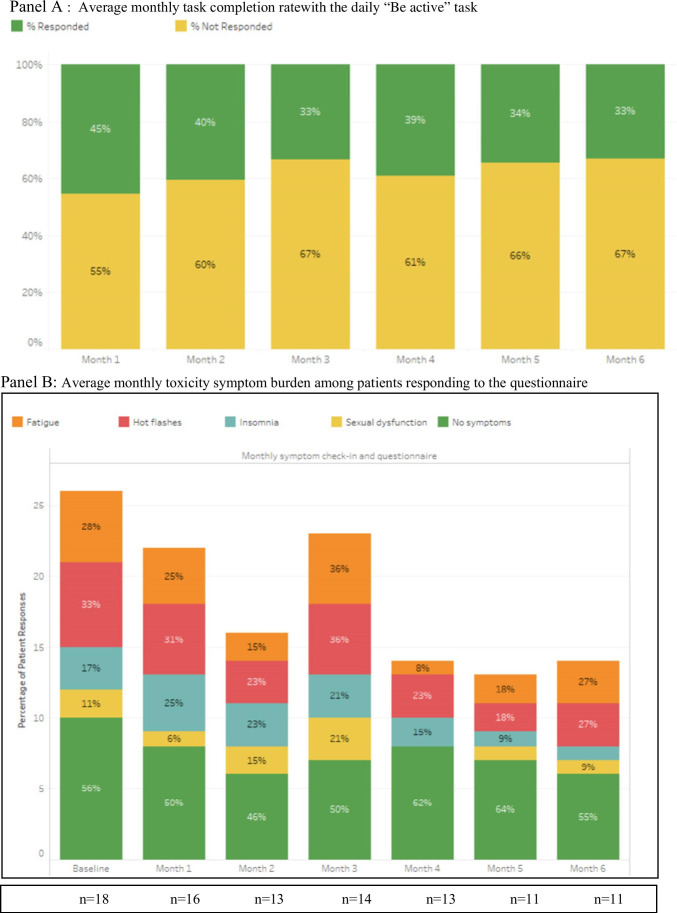
**A** Average monthly task completion rate with the daily “Be active” task. **B** Average monthly toxicity symptom burden among patients responding to the questionnaire. Note: *n* = # of responders. The monthly percentages may add to more than 100% due to patients who may report experiencing more than one symptom on the monthly questionnaire

The QOL PROMIS-29 questionnaire had a 6-month average completion rate of 62%, starting at 91% at baseline (*n* = 21), followed by 73% at month 1(*n* = 16), 52% at month 3 (*n* = 12), and 35% at month 6 (*n* = 8). The overall “Be active” daily task completion rate was 37% as assigned to all patients, with 22% of patients (*n* = 5) being compliant (completed this task ≥ 75% of the time). Completion of the activity task consistently decreased every month from enrolment, with the highest completion rate of 45% (14 days/month) during month 1 to 33% (10 days/month) during month 6 (*p* = 0.1975) (Fig. 2a). Two patients requested a change in the timing of the “Be active” reminder notification from early morning to the noon time, to make it more effective on reminding them of the task at a time when they are ready to exercise or have completed the exercise and they are ready to check off the task from their screen. This change was implemented at month 8 from study activation.

Engagement with the mindfulness education tasks at baseline was 86% for the Introduction to Mindfulness video, 65% for the Living in the Moment video, and between 30 and 38% for the body scan, meditation on the breath, on the body, and on the sounds, thoughts and emotions videos.

We did not receive any message directly from the study participants requesting changes to the frequency of task delivery or early discontinuation of the ICP.

### ePRO, BC recurrence symptoms, and QOL tracking and actions by ICP

The frequency of treatment-related toxicities, as tracked and similarly reported in both the ICP dashboard and patient EHR, was highest overall for hot flashes with an average of 27% of the responders endorsing this symptom (monthly range 18–33%), followed by fatigue in 22% (range 8–36%), insomnia 17% (range 9–25%), and sexual dysfunction 10% (range 0–21%) (Fig. 2b).

In response to patients self-reporting any degree of symptoms, app-embedded education materials were made available on-demand, and they were requested most frequently for sexual dysfunction at 60% (6 times requested, out of 10 reports of sexual dysfunction), followed by insomnia 35% (6 of 17), hot flashes at 33% (9 of 27), and fatigue 32% (7 of 22).

Of the 23 participants, 17% (*n* = 4) reported symptoms associated with BC recurrence at baseline, 9% (*n* = 2) at month 1 and month 3, and 4% (*n* = 1) at month 5. The most commonly reported symptoms were frequent headaches (reported 5 times) and persistent cough (reported 4 times). These were consistently reported in the ICP dashboard and the patient EHR, and each report appropriately resulted in an automatic message to the research In Basket.

The PROMIS QOL scores were available in the ICP dashboard for tracking and consistently reported in the patient EHR. Table 2 depicts the T scores of the subscales of the PROMIS questionnaire. There was no statistical difference in any of the PROMIS questionnaire subscales except for the Pain Intensity subscale which increased from an average of 1.3 at baseline to 1.5 at month 1, decreased to 0.6 at month 3, and then increased again to 1.7 at month 6 (*p* = 0.048). Two patients reported severe symptoms of anxiety and/or depression, and both resulted in automatic messages to the research In Basket, as intended.

**Table 2:** QOL PROMIS-29 T scores and pain scores

Subscale	Baseline		Month 1		Month 3		Month 6		*P* value for trend over time*	P value comparing baseline to month 6**
	N	Mean (SD)	N	Mean (SD)	N	Mean (SD)	N	Mean (SD)		
Physical functioning	22	53.6 (5.8)	15	53.1 (5.7)	14	55.4 (3.3)	10	51.5 (6.6)	0.2710	0.5949
Anxiety	22	52.7 (7.1)	15	51.6 (9.7)	14	50.6 (8)	9	46.7 (8.8)	0.2003	0.1475
Depression	22	47 (6)	15	49.1 (5.7)	14	49.5 (6.4)	10	46.8 (6.7)	0.1932	0.9350
Fatigue	22	47.2 (9.6)	15	47.1 (5.8)	14	46.9 (6.3)	10	48.6 (8.3)	0.8016	0.8457
Sleep disturbance	22	49.4 (6.4)	15	47.4 (6.6)	14	48.7 (5.6)	10	46.6 (11.3)	0.7350	0.4809
Social functioning	22	54.4 (6.3)	15	57 (8.2)	14	57.1 (6.8)	10	56.8 (6.9)	0.3342	0.0211
Pain interference	22	47.9 (7.5)	15	48.1 (7.6)	14	46.1 (6.2)	10	48.6 (6.2)	0.6283	0.5851
Pain intensity	22	1.3 (1.2)	15	1.5 (1.7)	14	0.6 (0.8)	10	1.7 (1.6)	0.0480	0.2443

^*^Repeated measures ANOVA

^**^Paired *t* test. This test *only* includes people who answered both the baseline and the month 6 questionnaires (*n* = 10 for all, anxiety: *n* = 9)

### Care team burden

The research In Basket received a total of 21 messages (average of 0.9 messages/patient) over the course of the study period. Of these, 10 were automatic messages generated when the patient was dismissed from the hospital, as the ICP was paused during the admission. In all cases, the ICP was restarted. Eleven of 253 symptom questionnaires (4.3%) generated a message that required care escalation to the patients and care team, including 2 related to severe symptoms of anxiety and/or depression and 9 for symptoms suspicious for cancer recurrence.

## Discussion

Telehealth and remote monitoring solutions may support symptom management and address some of the unmet needs of cancer survivors [17], [19], [20] . Our study team engaged patients and multidisciplinary members of the care team to develop a novel ICP to improve upon static survivorship care plans. In this pilot feasibility study, we demonstrated that the ICP may be ready for larger scale implementation in the oncology practice, but we also found the need for strategies to increase adoption and use, given the high engagement rate, but lower than predicted overall task completion rate over time.

Our 6-month average ePRO and BC recurrence assessment completion rates of 59% (range 48–78%), as well as the QOL questionnaire completion rate of 62% (range 35–91%), reflect the observed gradual decrease in response rate over time. Our response rates were higher than those of similar feasibility engagement study of a mobile health application intended for perioperative education of BC patients (reporting a 97% PROMIS response rate preoperatively, 24% postoperatively, and 15% at 3 months) [19] and similar to the monthly ePRO response rate in a large-scale implementation study involving cancer patients receiving active treatment at Mayo Clinic (personal communication, data not yet published) [28]. There is ample evidence that participation drops off quickly after enrolment in studies evaluating digital health solutions, which may be due to the time commitment involved, suboptimal user interfaces, loss of the novelty factor over time, and perceived lack of the effectiveness of the intervention for the needs of the participants [29], [30]. It is also feasible that cancer survivors specifically are feeling better through their post-treatment recovery and may feel less need for additional support over time.

Given our findings and the goal to develop an intervention that is impactful to the wellbeing of the cancer survivors and not cumbersome to both patient and care teams, we are planning to conduct focused group discussions with current ICP study participants to further understand facilitators and barriers to engagement from the perspectives of patients and lay caregivers, especially as those may change over time. We also intend to identify high and low impact ICP components and preferences for future content.

Furthermore, to assure a successful broad implementation of the ICP in clinical practice, we are planning a number of implementation strategies in feasibility trials, recommended by a group of experts in implementation strategies, such as (1) develop strategies with patients to encourage and problem-solve around adherence; (2) facilitate the formation of groups of providers, such as oncologists and primary care providers who care for BC survivors, to foster a collaborative learning environment and improve implementation of the ICP; (3) include actively engaged ICP patients and families in the implementation effort, by sharing the benefits of the tool with others in the community; and (4) provide ongoing consultation with one or more experts in the strategies used to support implementing the innovation [31].

In addition, given the lower than expected completion rate to the “Be active” task at 37%, possibly due to perceived low value or unclear instructions, and given the importance of physical activity and its correlation with BC survivor clinical outcomes[32], [33], in future iterations, we are planning to monitor physical activity by passively collecting data from smartphones or wearable devices (e.g., accelerometers, fitness trackers), to reduce patient burden. Offering regular feedback to patients regarding changes in symptoms and activity levels and designing an attractive user interface may also improve engagement, as suggested by developers of successful digital platforms [34].

While this was a feasibility study, it is worth noted that the most frequently requested education was for sexual dysfunction (60% of instances of sexual dysfunction). This may suggest a difference in preferred format for this topic or a gap in addressing the sexual dysfunction topic during clinical encounters, a sensitive topic difficult to discuss and often avoided by both patients and providers [35, 36]. The frequency of education requests in response to reported symptoms for the other treatment-related toxicities (hot flashes, fatigue, and insomnia) was relatively low (32—35%). It is unclear why patients did not request educational content more often, and we plan to learn more from the patient satisfaction surveys and qualitative focused group discussions. These learnings will be incorporated into future iterations of the education materials and targeted symptoms.

We also demonstrated that the impact of the ICP on the clinical care team was minimal, with 21 total messages generated over the 9-month study period, only 11 of which were escalated due to their association with possible clinical concerns requiring patient assessment and, when warranted, additional workup. The care escalation burden was 4.3%, which was within our hypothesized number of < 10%. This finding is important as many providers and nurses are concerned that remote monitoring tools will increase messages and calls, and as such are reluctant to adopt them in clinical practice.

No patient has yet completed the full 12-month ICP; therefore, participant satisfaction data is not available. As part of the next iteration, we are planning to add a satisfaction survey at month 6, to facilitate more agile changes to the ICP based on patients’ feedback.

To our knowledge, this is among the first studies reporting an app-based care plan to remotely monitor patients with ePROs and questionnaires built and embedded directly into both patient- and provider-facing EHR tools. The benefit of this is having the patient-facing care plan embedded within their health care app (Mayo Clinic mobile app and patient portal account), and the patient-generated health data and escalation messages immediately available to the care teams without need for a separate, remote monitoring, web-/app-based system, and dashboard. In a published overview of digital platforms used in cancer care, the authors make a strong case for seamless integration of digital health technologies into the EHR, in order to maximize their potential to capture ePROs that can improve patient QOL and outcomes while concurrently reducing costs and workflow inefficiencies with separate systems [37]. All these factors may influence the patient and care team experience and lend support to collect additional data on patient and provider experience.

In addition to planned iterations of this ICP to enhance existing and create new functional capabilities, we also aim to ensure that a more diverse patient population is offered access to the ICP in keeping with the Patient-Centered Outcomes Research Institute vision of representation of all stakeholders in the research question.

Finally, it is feasible that the established framework for this ICP can be easily adapted to create new care plans to support survivors of other cancers. Additionally, some of the existing content is likely repeatable given that many toxicities and symptoms of recurrence are common among survivors. This affirms the scalable nature of this digital platform and approach. We plan to conduct larger scale assessment of both effectiveness and implementation outcomes (e.g., acceptability, fidelity, sustainability) during expansion into new clinical domains. The results of this study and our upcoming mixed methods inquiry with patients and caregivers will inform the theoretical approach for subsequent larger scale assessment of implementation, e.g., those focused on individual behavior change or structural barriers to implementation.

Our study was limited to a single cancer tumor group and conducted at a single institution, making its findings difficult to generalize. Additionally, the study is also limited by the cohort being predominantly non-Hispanic white, married women. These limitations will be addressed in future studies of the ICP, which will be offered at other more urban Mayo Clinic sites (Jacksonville, Florida, and Phoenix, Arizona), as well as rural sites within the community-based Mayo Clinic Health System throughout the Midwest. With expansion of the ICP to these other sites, and given the application is available to other institutions that utilize Epic as their EHR vendor, we aim to understand its feasibility and impact in a more diverse patient populations and clinical settings. More male-specific educational content for management of sexual dysfunction will be required to extend the full ICP offering to male BC survivors as well.

Implementation of this ICP occurred in August 2020, coinciding with the “fall surge” of COVID-19 cases in the Midwestern states. It is difficult to interpret how the COVID-19 pandemic impacted engagement with this app-based care plan. Just like other aspects of healthcare and lifestyle changes were impacted by the pandemic, some people may have been more prone to engage with a new application to keep them connected with their care team when in-person visits required travel and increased exposure and to create a sense of control in their life when events of the COVID-19 pandemic felt uncontrollable. Other people may have been less prone to engage with it given feelings of anxiety and distrust around healthcare. We will be tracking and comparing the engagement between the cohorts recruited in 2020 versus 2021 to better understand this.

## Conclusion

Implementation of an EHR-integrated, app-based ICP in the breast cancer survivorship practice is feasible and minimally burdensome for care teams. The app could be used for remote monitoring and to track changes in activity, symptoms, and QOL; however, more work is needed to enhance patient engagement with the tool over time.

## Supplementary Information

Below is the link to the electronic supplementary material.

Supplementary file1 (PDF 110 kb)

## Data Availability

The datasets generated during and/or analyzed during the current study are available from the corresponding author on reasonable request.
