# Supplementary material for: Pilot implementation to assess the feasibility and care team impact of an app-based interactive care plan to remotely monitor breast cancer survivors
Source: J Cancer Surviv. 2022 Feb 2;16(1):13–23. doi: 10.1007/s11764-021-01136-1 (PMC8809246; doi:10.1007/s11764-021-01136-1)

Supplemental Figure 1: ICP Content comprised of scheduled tasks including Reminders, Questionnaires, and Education.

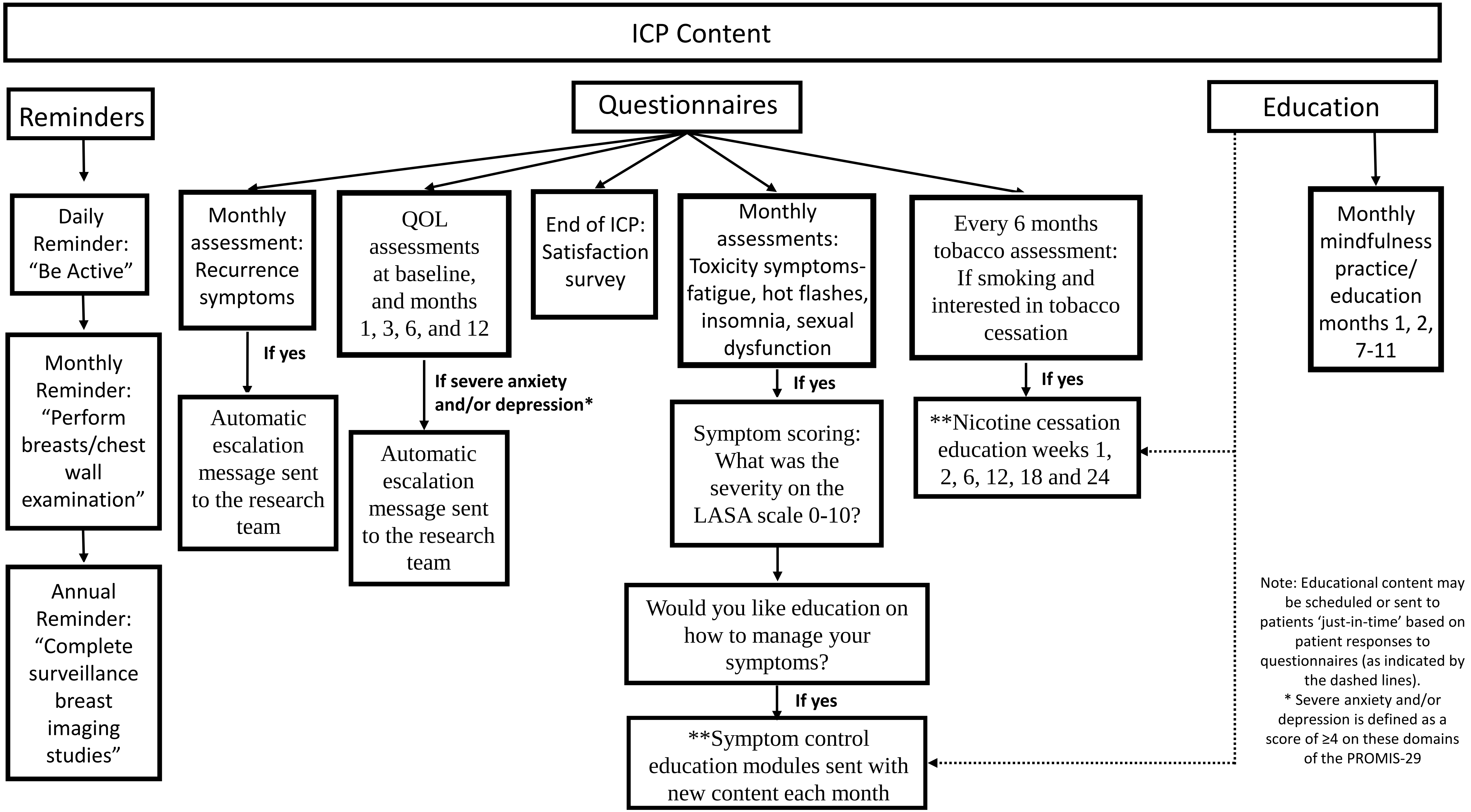

Supplement: Supplementary file 1 — Supplementary file1 (PDF 110 kb) [file 11764_2021_1136_MOESM1_ESM.pdf]
